# Prevalence and Risk Factors of Parasitic Gastrointestinal Nematode Infections of Donkeys in Southern Ethiopia

**DOI:** 10.1155/2024/3073173

**Published:** 2024-04-15

**Authors:** Isayas Asefa Kebede, Haben Fesseha Gebremeskel, Tamench Bandaw, Abrahim Dawed Ahmed

**Affiliations:** ^1^School of Veterinary Medicine, Ambo University, P.O. Box 19, Guder, Ethiopia; ^2^School of Veterinary Medicine, Wolaita Sodo University, P.O. Box 138, Wolaita Sodo, Ethiopia; ^3^Offa District Veterinary Clinic Animal Health Expert, Offa, Wolaita Zone, Ethiopia; ^4^Ethiopian Agriculture Authority, Eastern Branch, Dire Dawa, Ethiopia

## Abstract

Gastrointestinal (GIT) parasites cause sickness and mortality in working donkeys, reducing their productivity. A cross-sectional study was done in the Damot Gale district of southern Ethiopia from November 2020 to June 2021 to determine the frequency of donkey GIT nematode parasite infection and to examine its related risk factors. Overall, 514 simple randomly selected donkeys from peasant associations were sampled for the coprological examination of gastrointestinal nematode infection. The flotation technique was employed to identify parasite eggs in feces. The total prevalence of parasitic gastrointestinal nematodes was 71.79% (95% CI: 67.73-75.52). The most prevalent nematodes were *Strongyles* (37.74%), *Parascaris equorum* (11.28%), *Strongyloides* (7.20%), and combined infections of *Strongyles* and *Parascaris* (14.01%) and *Strongyles* and *Strongyloides* (1.56%). The association between the prevalence of parasitic GIT nematode infections and body conditions score was statistically significant (*p* < 0.05). Comparatively, donkeys with semi-intensified systems were five times (OR = 5.36) and those with medium body condition were twice (OR = 1.94) had a higher risk of infection than donkeys with intensive systems and good body condition scores, respectively. In conclusion, the current study indicated that gastrointestinal nematode parasites of donkeys are highly prevalent in the study area. Thus, regular deworming, proper housing, and feeding management were recommended to improve the health and productivity of donkeys in the research area.

## 1. Introduction

The domestic donkey (*Equus asinus*), a member of the Equidae family, was domesticated some 5000 years ago in Africa and has since spread around the world [1]. The donkey is supposed to be a descendant of the Nubian assailant. The global donkey population is estimated to be about 44 million, with half living in Asia, a quarter in Africa, and the remainder primarily in Latin America [2]. With roughly 7.4 million donkeys, Ethiopia has the world's third-largest equine population [3].

In 98 percent of Africa's semiarid zones, they are utilized for work, breeding, milking, and meat production [1, 4]. Donkeys are a wonderful alternative in locations where other terrains, such as mountains and cities with narrow streets, make goods transportation problematic, and they are also important in agricultural activities [5]. They are retained and used for energy and soil fertility in ploughing, cultivation, and threshing, as well as manure [6]. Donkeys are noted for their power and toughness. They are, nevertheless, vulnerable to parasite infections [7, 8].

Gastrointestinal parasite infection is a major health and welfare issue for working donkeys, limiting the profitability of donkey performance all over the world [9]. Some GIT parasites are aggressive bloodsuckers, such as Strongyles, and produce varying degrees of harm depending on the species and numbers present, as well as the nutritional and immunological health of the equids [10]. They cause a severe health danger, producing poor body condition, lower power output, decreased productivity, and a short lifetime. According to existing information, gastrointestinal helminths are the leading cause of early death in donkeys [6, 11].

More than 150 different helminth parasites can infect donkeys. The most common and harmful helminth parasites include large and small *strongyles*, roundworms, tapeworms, lungworms, pinworms, threadworms, and bots. The most dangerous nematodes of health concerns are probably large and small *strongyles*, roundworms, and tapeworms [12–14]. Large *strongyles* (*Strongyles vulgaris*, *Strongylus equinus*, *Strongylese edentatus*, and *Triodontophorus* species), *Parascaris equorum*, *Oxyuris equi*, and, to a lesser extent, other small *strongyles* (*cyathostomins*) are the most prevalent intestinal nematodes found in equids. Equid intestinal nematodes have a similar life cycle. Clinical sickness is caused not only by the presence of the adult parasite in the bowel but also by larval migration in the colon and other organs, most notably the circulatory system [12, 15].

GIT parasites are frequent in poorer countries where food and hygiene are often poor, and donkeys have significant problems [10, 14, 16, 17]. In Ethiopia, where health care is poor, particularly for equines, the prevalence, species composition, and epidemiology of GIT parasites affecting donkeys have not been adequately explored [16–18].

Donkeys are commonly used as working animals, even though they are susceptible to several diseases and are usually asymptomatic carriers [19]. Aside from a few studies in other parts of Ethiopia, no previous research on gastrointestinal (GIT) nematodes of donkeys in the Damot Gale district has been undertaken. As a result, the current study is aimed at assessing the prevalence and risk factors for gastrointestinal nematode infections in donkeys in the study area.

## 2. Materials and Methods

### 2.1. Study Area

**Figure 1:**
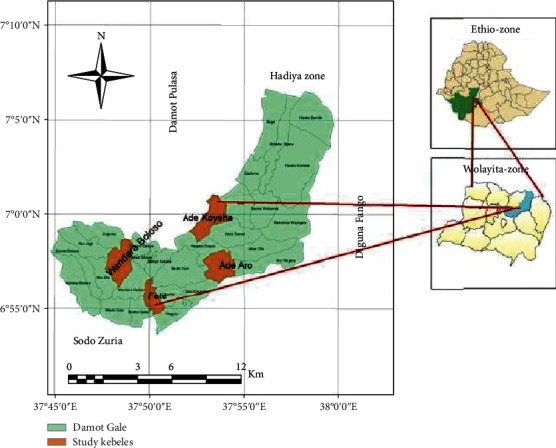
Map of the study area.

The research was carried out in the Damot Gale district, which is located in northern Ethiopia (Figure 1). The research area's altitudinal ranges from 1,200 to 2,950 meters above sea level, with an average annual rainfall of 900-1,400 mm. Damot Gale Woreda is bounded on the southwest by Sodo Zuria, on the northwest by Boloso Sore and Damot Pulasa, on the north by the Hadiya Zone, on the east by Diguna Fango, and on the south by Damot Weyde. The area has bimodal rainfall, with a short rainy season from mid-January to April and a lengthy wet season from June to mid-October. The average lowest and maximum temperatures were 12 and 27°C, respectively. The Woreda is astronomically positioned between 6°5500 and 7°1000N latitude and 37°450 and 38°0E longitude.

### 2.2. Study Animals

The study animals were indigenous donkey breeds from the Damot Gale district, with different body states, ages, sex, management, and provenance. The study's target group was working donkeys that had not been dewormed in the previous months and the deworming history was obtained from donkeys' owners. Donkeys of all ages, sexes, and body condition groups were included. The age of the study donkeys was determined based on dentition patterns (the twelve front incisors, the shape of the permanent upper corner of the incisors and table of the central incisors, and the disappearance of the enamel ring) [20] and classified as young (5 years), adult (5-10 years), and old (>10 years). The working donkey's body condition scoring (BCS) was assessed based on the deposition of body fat in different locations by separate assessment of the neck, back, ribs, pelvis, and rump [21].

### 2.3. Study Design

A cross-sectional study was conducted in the Damot Gale district from November 2020 to June 2021 to determine the prevalence and associated risk factors of the GIT nematode parasite of donkeys. Donkeys in the study areas were picked at random for GIT parasite investigations, independent of their age, gender, physical condition, or color. On daylight from several communities in the district, the sampling technique was carried out at the field level, market, homestead, and surrounding water point locations.

### 2.4. Sample Size Determination

The sample size for the study was calculated using Thrusfield et al.'s formula [22]. Because there had been no previous report on the prevalence of the parasites in the study area, the sample size was determined using a 50% projected prevalence for gastrointestinal helminths. In addition, the required absolute precision of 5% and confidence level of 95% were used.

(1) $$\begin{array}{c} n=Z^{2}\times \frac{P\exp\left(1-P\exp\right)}{d^{2}}, \end{array}$$

where *n* is the required sample size, *Z* is the confidence level (1.96), *P*exp is the expected prevalence (50%), and *d*^2^ is the desired absolute precision (0.05).

As a result, the minimum sample size necessary in the target area was 384; however, a 514-sample size was employed in the study area to boost precision.

### 2.5. Sampling Method and Sample Collection

The study animals were chosen using a simple random sampling procedure. In the research area, 514 fecal samples were collected from donkeys. The sample was collected in the daytime from selected kebeles. The sampling procedure was carried out at the market, field level, homestead, and around water point locations after donkeys were physically restrained. After restraint, approximately 10 grams of fecal samples [18] were obtained with gloved hands directly from the rectum or recently defecated excrement and placed in screw-cupped bottles and carefully labeled. The samples were then delivered in an ice box to the Sodo regional veterinary laboratory's veterinary parasitology section. When rapid processing was not possible, the samples were held in refrigerators at 4°C; however, the majority of samples were processed within 48 hours.

Floatation procedures were used for fecal testing [23, 24]. Briefly, 3 grams of feces were measured, crushed in a mortar and pestle, and placed into a glass beaker, followed by the addition of 40 ml of flotation fluid (the flotation fluid was made of supersaturated sodium chloride (NaCl) solution). The mixture was stirred continually using a glass rod. The dissolved suspension was then filtered into a separate beaker using a tea strainer. The suspension was then moved to another test tube until a meniscus formed at the top, after which a cover slip was gently placed over it and left to stand for 20 minutes. Finally, the supernatant fluid-adhered coverslip was carefully removed from the test tube, transferred to a microscopic slide, and inspected under a microscope. The eggs of parasites were recognized using a compound microscope (Magnus Binocular Microscope, India, 4x-100x) (10x objectives) [25, 26].

### 2.6. Data Management and Analysis

The fecal examination data were coded and entered into a Microsoft Excel spreadsheet 2016 before being analyzed using STATA 13 software (Stata Corp LP, College Station, Texas). To assess the prevalence in percentages, a descriptive analysis was undertaken. The significance and degree of relationship between risk variables and gastrointestinal nematode parasites were determined using univariate and multivariate logistic regressions. Furthermore, using logistic regression (reporting odds ratio), the impacts of specific possible risk factors (animal origin, age, sex, body condition, and management) on GIT nematode infection were investigated. The risk factors with a *p* value ≤ 0.25 in univariable analyses were selected for multiple logistic regression analyses. The final multiple logistic regression models were built using a backward elimination approach. The threshold of significance was regarded when the *p* value was less than 0.05 for variables with a significant odds ratio (OR) value at a 95% confidence interval.

## 3. Results

### 3.1. Distributions of GIT Nematode Parasite in the Study Area

**Figure 2:**
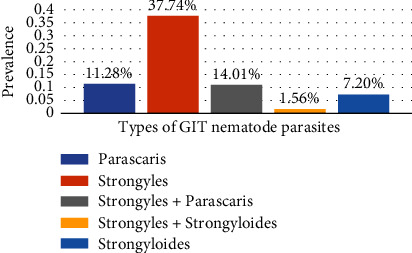
Distributions of parasites among positive results in the study area.

*Strongyles* were found in 37.74% of the 514 total fecal samples obtained from donkeys and tested for the presence of different gastrointestinal nematode parasites, followed by *Parascaris* (11.28%) and *Strongyloides* (7.20%) (Figure 2).

### 3.2. Prevalence of Gastrointestinal Nematode Infection with Their Potential Risk Factors

According to the present study, the highest prevalence of GIT nematode parasites was recorded in Fate kebele, female, adult, semi-intensive farming systems, and medium-body condition donkeys, with a prevalence of 76.23%, 73.08%, 76.83%, 83.33%, and 79.16%, respectively (Table 1).

### 3.3. Univariable Logistic Regression Analysis

A univariable logistic regression analysis was also carried out to determine the strength of the association between risk factors and gastrointestinal parasite infection. The univariable logistic regression analysis of the risk factors demonstrated a significant (*p* < 0.05) association between the occurrence of GIT nematode infections and the risk factors of body condition, management, and age. Donkeys with medium body conditions were twice as likely (OR = 2.15) to be infected as donkeys with good body conditions. Similarly, semi-intensified and extensively managed donkeys were five (OR = 5.76) and two (OR = 2.47) times, respectively, more likely to be infected than intensified donkeys (Table 2).

### 3.4. Multivariable Logistic Regression Analysis

Those potential risk factors (origin, age, management, and BSC) with *p* values less than 0.25 were subjected to multivariable logistic regression analysis using the backward elimination technique, and the final model was developed. Thus, management and body condition score was the only risk factor associated with the GIT nematode infections, and hence, it was statistically significant (*p* < 0.05). Donkeys that are kept semi-intensively and extensively are 5.3 and 2.67 times more likely to be infected than donkeys that are intensively managed (Table 3). Moreover, the Hosmer-Lemeshow goodness-of-fit test suggested that the model fit the data (HL*χ*^2^ = 161.24; Prob > *χ*^2^ = 0.2870) and multicollinearity found not to violate the assumption (AUC = 71.90%).

## 4. Discussion

Intestinal parasitism has a direct impact on the health and productivity of draft donkeys, resulting in a decline in draft performance and, ultimately, income for the owner and community [9, 27, 28]. The overall prevalence of GIT parasites was 71.79% (95% CI: 67.73-75.52) in the current study, which is similar to previous publications that indicated 70.4% in the south Wollo zone [29]. However, this figure was lower than recent reports of 88.2-97.2% in different locations in Ethiopia [27, 28, 30] and elsewhere around the world [31, 32]. This study's findings, on the other hand, were greater than the previously reported frequency of 37.48% of donkeys in South Darfur state [33]. This disparity could be attributable to variations in sampling time, as seasonality influences the occurrence of the parasites. Furthermore, donkeys' access to free-range pastureland increases the likelihood of ingesting the eggs and larvae of a wide range of GIT parasites. The availability of veterinary services, donkey deworming practices, and the feeding of these animals with supplementary feed all have an impact on the occurrence.

Besides, the coproparasitological approaches may be ascribed to the discrepancy in findings (the chemical employed for flotation and sample preservation, etc.). Compared to others, our research season was just semidry. In this study, donkeys that were not dewormed and did not have parasites may have had immune systems (resilience-resistance) and particular attention (feeding and watering) supplied by their owners.

In the current study, the prevalence of *strongyles* was 37.74%, which is lower than the findings of Ayele et al. [34] in Dugda Bora districts, Zerihun et al. [11] in Sululta and Gefersa, Yoseph et al. [35] in Wenchi, and Mulate [36] in highlands of Wollo province, who reported a prevalence of 100%, and elsewhere Wannas et al. [37] in Al Diwaniyah Governorate and Seri et al. [38] in Sudan reported a prevalence of 99.15%. Conversely, the current findings were higher than the report of Worku and Afera [39] in Kombolcha town and Mathewos et al. [27] in Hawassa, who reported a prevalence of 32.6% and 6.07%, respectively. These discrepancies may be due to disparities in topographical character and abuse, a lack of coverage for donkey health treatment, and improper donkey husbandry techniques. Parasite survival rates vary dramatically depending on the season and temperature of the agroecology. Strongyles are common, especially in hot and humid areas. Strongyloides infective larvae are not coated, leaving them exposed to extreme weather. Warmth and dampness, on the other hand, encourage development and allow a large number of infectious stages to develop. The reservoir of larvae in their dams' tissues is a second important source of infection [23].

In this study, the prevalence of *P. equorum* was 11.28%, which is consistent with the 11.2% reported by Getahun and Kassa [40] in Tenta Woreda and the 15.1% reported by Asefa and Dulo [41] in Bishoftu town. However, this is higher than the 6.4% reported by Gebreyohans et al. [42] in and around Mekelle and lower than the 26.2% reported by Tesfu et al. [43] in Hawassa town. These variations in prevalence could be attributed to the length and season of the study period, the agroecology of the study area, the relatively low numbers of these parasites in the pasture, and the use of parasite control programs. Furthermore, the biology of *P. equorum*, such as the high fecundity of adult female parasites, the egg's extreme resistance in the environment and its long-term presence, and the sticky nature of the outer shell, facilitates the passive spread of eggs and leads to prevalence variations [12, 23].

In this study, the prevalence of Strongyloides was 7.20%, which is equal to 9.5% reported by Getahun and Kassa [40] in Tenta Woreda. However, the prevalence was higher than that reported by Gebreyohans et al. [42] in Mekelle and the suburbs but lower than that reported by Mathewos et al. [27] in Hawassa, where the prevalence was 2% and 50.0%, respectively. The intensity of parasite infections may be influenced by differences in the study period, agroecology, and veterinary services, such as infrastructure quality.

According to the current study, the prevalence was significantly greater in animals with medium body conditions, at 79.16%, than in donkeys with poor and good body condition ratings, at 63.90% and 69.76%, respectively. The occurrence of GIT nematodes has been connected to risk variables such as management systems and body condition (*p* < 0.05). The reason behind this is unclear and needs further studies. This contradicts the findings of Ayele et al. [34], who reported that animals with medium body conditions had a lower prevalence of helminth parasites than animals with poor body conditions. Infection is two (OR = 1.94) times more frequent in medium body conditions than in good body conditions. This could be due to malnutrition or other concurrent bacterial and parasitic infections, which result in a weakened immune response to the parasites' infective stage. The production of eggs by nematodes is also dependent on the host's immune condition, as innate immunity can limit egg production even if the animal is in perfect health. Farmers can utilize the body condition score to identify donkeys in need of anthelmintic treatment [18, 34].

For this study, semi-intensive, intensive, and extensive management systems were widely used in the study area, which included those allowed to graze with some extra feed supplements at home, those kept on different feed supplementations at home during and/or after work, and those reared in outdoor grazing without additional feed supplements, respectively. In terms of the management system, there was a very statistically significant difference (*p* < 0.05). The prevalence of helminth infection was greater (83.33%) in semi-intensive systems, while it was 46.46% and 68.21% in extensive and intensive management systems, respectively. Semi-intensified donkeys were five times (OR = 5.36) more likely to be infected than intensified donkeys in this study. This meant that semi-intensified donkeys were more likely to be infected by the parasite. This could be because semi-intensified animals are immune-compromised as a result of the high workload. Similarly, the extensively handled donkeys were two (OR = 2.51) times more likely to be infected than intensified donkeys. In the study area, animals kept in semi-intensive management systems used for packing, transport, and carting had higher workloads than animals kept in intensive systems, which could be explained by the difference in management care provided to these groups of animals. However, the intensively kept donkey is given special care, such as deworming and supplementary feeding, and has less opportunity to graze, lowering their risk of infection compared to others.

### 4.1. Limitations of This Study

The current study did not examine the seasonal distributions of GIT nematode parasites in donkeys. Furthermore, fecal culture and larval identifications for several parasite species identifications were not attempted in this investigation, and egg counting was not undertaken to establish severity. This is owing to the limited study period and the fact that some needed equipment was not working.

## 5. Conclusion

In conclusion, GIT nematode infestation was shown to be common in donkeys, with a prevalence of 71.46% in male donkeys and 73.08% in female donkeys. This study discovered that gastrointestinal parasites such as *Strongyle*, *Parascaris equorum*, and *Strongyloides* are prevalent in donkeys in the study area. Donkeys with medium body conditions had greater parasite levels than good-conditioned animals. The most significant risk factors for gastrointestinal nematode infection are management and body condition. Finally, donkey owners should be taught about the factors that predispose their donkeys to parasitic infection as well as strategies to mitigate the negative impacts of parasitic infection. To lessen the burden of parasite infection in the research area, an effective parasite control approach should be established.

## Figures and Tables

**Table 1:** Prevalence of nematode parasite with association of risk factors.

Variables	Category	No. of examined	No. of positive	Prevalence (%)	95% CI
Origin	Wandara Boloso	110	75	68.18	58.86–76.24
	Fate	122	93	76.23	67.82–82.98
	Ade Koyesha	138	94	68.11	59.84–75.38
	Ade Aro	144	107	74.30	66.50–80.81

Sex	Male	410	293	71.46	66.88–75.64
	Female	104	76	73.08	63.69–80.76

Age	Young	149	95	63.75	55.69–71.11
	Adult	272	209	76.83	71.42–81.48
	Old	93	65	69.89	59.76–78.39

Management system	Semi-intensive	264	220	83.33	78.31–87.37
	Intensive	99	46	46.46	36.83–56.37
	Extensive	151	103	68.21	60.32–75.17

Body condition score	Poor	129	90	69.76	61.25–77.10
	Medium	216	171	79.16	73.20–84.08
	Good	169	108	63.90	56.35–70.82

**Table 2:** Univariable logistic regression analysis of risk factors associated with GIT nematode infections.

Variables	Category	Prevalence (%)	OR	95% CI	*p* value
Origin	Ade Koyesha	68.11	Ref	—	—
	Fate	76.23	1.49	0.84-2.67	0.172
	Wandara Boloso	68.18	1.00	0.58-1.707	0.991
	Ade Aro	74.30	1.35	0.78-2.34	0.284

Sex	Male	71.46	Ref	—	—
	Female	73.08	1.08	0. 67-1.76	0.744

Age	Young	63.75	Ref	—	—
	Adult	76.83	1.89	1.22-2.92	0.004
	Old	69.89	1.32	0.76-2.30	0.327

Management system	Intensive	46.46	Ref	—	—
	Extensive	68.21	2.47	1.47-4.17	0.001
	Semi-intensive	83.33	5.76	3.46-9.60	<0.001

Body condition score	Good	63.90	Ref	—	—
	Medium	79.16	2.15	1.36-3.38	0.001
	Poor	69.76	1.30	0.79-2.13	0.289

**Table 3:** Multivariable logistic regression analysis of risk factors associated with GIT nematode infections.

Variables	Category	Prevalence (%)	OR	95% CI	*p* value
Origin	Ade Koyesha	68.11	Ref	—	—
	Fate	76.23	1.39	0.75-2.61	0.298
	Wandara Boloso	68.18	0.87	0.48-1.55	0.639
	Ade Aro	74.30	1.12	0.62-2.06	0.699

Age	Young	63.75	Ref	—	—
	Adult	76.83	1.60	0.98-2.63	0.059
	Old	69.89	1.33	0.74-2.42	0.341

Management system	Intensive	46.46	Ref	—	—
	Extensive	68.21	2.66	1.52-4.66	0.001
	Semi-intensive	83.33	5.30	3.13-8.99	<0.001

Body condition score	Good	63.90	Ref	—	—
	Medium	79.16	1.96	1.21-3.19	0.007
	Poor	69.76	1.60	0.93-2.74	0.086

OR = odds ratio; Ref = referent category; CI = confidence interval.

## Data Availability

All the datasets generated or analyzed during this study are included in this manuscript.

## References

[1] Burden F., Thiemann A. (2015). Donkeys are different. *Journal of Equine Veterinary Science*.

[2] Fielding D., Starkey P. (2004). *Donkeys, People, and Development. A Resource Book of the Animal Traction Network for Eastern and Southern Africa (ATNESA)*.

[3] Central Statistical Agency Livestock and Livestock Characteristics (2016). *Agricultural sample survey*.

[4] Tedla M., Abichu B. (2018). Cross-sectional study on gastro-intestinal parasites of equids in south-western Ethiopia. *Parasite Epidemiology and Control*.

[5] Mezgebu T., Tafess K., Tamiru F. (2013). Prevalence of gastrointestinal parasites of horses and donkeys in and around Gondar town, Ethiopia. *Open Journal of Veterinary Medicine*.

[6] Gebrewold A., Tegegn A., Yami A. (2004). Research needs of donkey utilization in Ethiopia. *Donkeys, People and Development. A Resource Book of the Animal Traction Network for Eastern and Southern Africa*.

[7] Hosseini S. H., Meshgi B., Eslami A., Bokaei S., Sobhani M., Agha E. S. R. (2009). *Prevalence and biodiversity of helminth parasites in donkeys (Equus asinus) in Iran*.

[8] Chitra R., Rajendran S., Prasanna D., Kirubakaran A. (2011). *Influences of age on the prevalence of parasitic infections among donkeys in Erode district, Tamilnadu, India*.

[9] Fesseha H., Aliye S., Mathewos M., Nigusie K. (2022). Prevalence and risk factors associated with donkey gastrointestinal parasites in Shashemane and suburbs, Oromia region, Ethiopia. *Heliyon*.

[10] Mohammed Jajere S., Rabana Lawal J., Mohammed Bello A., Wakil Y., Aliyu Turaki U., Waziri I. (2016). Risk factors associated with the occurrence of gastrointestinal helminths among indigenous donkeys (*Equus asinus*) in northeastern Nigeria. *Scientifica*.

[11] Asefa Z., Asefa Z., Kumsa B. K. B. (2011). *Endoparasites of donkeys in Sululta and Gefersa districts of central Oromia, Ethiopia*.

[12] Radostits O. M., Gay C. C., Hinchcliff K. W., Constable P. D. (2007). *Veterinary Medicine: A Textbook of the Diseases of Cattle, Horses, Sheep, Pigs, and Goats*.

[13] Saeed M. A., Beveridge I., Abbas G. (2019). Systematic review of gastrointestinal nematodes of horses from Australia. *Parasites & Vectors*.

[14] Sazmand A., Bahari A., Papi S., Otranto D. (2020). Parasitic diseases of equids in Iran (1931–2020): a literature review. *Parasites & Vectors*.

[15] Regassa F., Dhuguma R., Sorry T., Bzunesh M. (2005). Prevalence of equine gastrointestinal parasites in western highlands of Oromia. *Bulletin of Animal Health and Production Africa*.

[16] Enigidaw S., Assefa A., Mekonnen N., Belete S. (2015). Prevalence of gastrointestinal nematode parasitic infections of horses and donkeys in and around Kombolcha town. *American-Eurasian Journal of Scientific Research*.

[17] Admassu B., Shiferaw Y. (2011). *The Brooke*.

[18] Beriso G., Tesfaye Z., Fesseha H., Asefa I., Tamirat T. (2023). Study on gastrointestinal nematode parasite infections of donkey in and around shone town, Hadiya zone, southern Ethiopia. *Heliyon*.

[19] Kumar S., Kumar R., Sugimoto C. (2009). A perspective on Theileria equi infections in donkeys. *Japanese Journal of Veterinary Research*.

[20] Elisabeth D. S. (2008). *The Professional Hand Books of the Donkey*.

[21] Vall E., Ebangi A. L., Abakar O. (2003). A method for estimating body condition score (BCS) in donkeys. *Working Animals in Agriculture and Transport*.

[22] Thrusfield M., Christley R., Brown H. (2018). *Veterinary Epidemiology*.

[23] Taylor M. A., Coop R. L., Wall R. (2015). *Veterinary Parasitology*.

[24] Hendrix C. M., Robinson E. D. (2022). *Diagnostic Parasitology for Veterinary Technicians-E-Book*.

[25] Foreyt W. J. (2013). *Veterinary Parasitology Reference Manual*.

[26] Zajac A. M., Conboy G. A., Little S. E., Reichard M. V. (2021). *Veterinary Clinical Parasitology*.

[27] Mathewos M., Girma D., Fesseha H., Yirgalem M., Eshetu E. (2021). Prevalence of gastrointestinal helminthiasis in horses and donkeys of Hawassa District, southern Ethiopia. *Veterinary Medicine International*.

[28] Debere D., Debere D., Muktar Y. M. Y., Shiferaw S. S. S., Belina D. B. D. (2018). *Internal parasites of equines and associated risk factors in and around Guder town, West Shewa, central Ethiopia*.

[29] Regassa A., Yimer E. (2013). Gastrointestinal parasites of equine in south Wollo zone, northeastern Ethiopia. *Global Veterinaria*.

[30] Berhanu T., Ibrahim N., Deressa B., Tolosa T. (2014). Prevalence of helminth parasites of horses in and around Hawassa town, southern Ethiopia. *Acta Parasitological Globalis*.

[31] Upjohn M. M., Shipton K., Lerotholi T., Attwood G., Verheyen K. L. P. (2010). Coprological prevalence and intensity of helminth infection in working horses in Lesotho. *Tropical Animal Health and Production*.

[32] Valdéz-Cruz M. P., Hernández-Gil M., Galindo-Rodríguez L., Alonso-Díaz M. Á. (2013). Gastrointestinal nematode burden in working equids from humid tropical areas of central Veracruz, Mexico, and its relationship with body condition and haematological values. *Tropical Animal Health and Production*.

[33] Sawsan M., Hassan T., Seri H. I., Zolain Hidaia B. Field investigation of gastrointestinal nematodes in horses and donkeys in South Darfur State, Sudan.

[34] Ayele G., Feseha G., Bojia E., Joe A. (2006). Prevalence of gastro-intestinal parasites of donkeys in Dugda Bora district, Ethiopia. *Livestock Research for Rural Development*.

[35] Yoseph S., Feseha G., Abebe W. (2001). Survey on helminthosis of equines in Wenchi. *Journal of the Ethiopian Veterinary Association*.

[36] Mulate B. (2005). Preliminary study on helminthosis of equines in south and north Wollo zones. *Journal of Veterinary Association*.

[37] Wannas H. Y., Dawood A., Gassem A. (2012). Prevalence of gastro-intestinal parasites in horses and donkeys in Al Diwaniyah governorate. *Al-Qadisiyah Journal of Veterinary Medicine Sciences*.

[38] Seri H. I., Hassan T., Salih M. M., Abakar A. D. (2004). *A survey of gastrointestinal nematodes of donkeys (Equus asinus) in Khartoum State, Sudan*.

[39] Worku S., Afera B. (2012). Prevalencia de nematodos en equinos en y alrededor del sur de Wollo Kombolcha, Ethiopia. *REDVET. Revista Electrónica de Veterinaria*.

[40] Temesgen K. G., Tihune Z. K. (2017). Prevalence and species of major gastrointestinal parasites of donkeys in Tenta Woreda, Amhara Regional State, Ethiopia. *Journal of Veterinary Medicine and Animal Health*.

[41] Asefa S., Dulo F. (2017). A prevalence of gastro-intestinal nematode parasitic infections in horses and donkeys in and around Bishoftu town, Ethiopia. *Middle-East Journal of Applied Sciences*.

[42] Gebreyohans A., Abrhaley A., Kebede E. (2017). Prevalence of gastrointestinal helminths of donkey in and around Mekelle. *National Science*.

[43] Tesfu N., Asrade B., Abebe R., Kasaye S. (2014). Prevalence and risk factors of gastrointestinal nematode parasites of horse and donkeys in Hawassa town, Ethiopia. *Journal of Veterinary Science & Technology*.

